# Towards Characterization of Skin Melanoma in the Clinic by Electron Paramagnetic Resonance (EPR) Spectroscopy and Imaging of Melanin

**DOI:** 10.1007/s11307-023-01836-3

**Published:** 2023-06-30

**Authors:** Mohammad Wehbi, Evelyne Harkemanne, Lionel Mignion, Nicolas Joudiou, Isabelle Tromme, Jean-François Baurain, Bernard Gallez

**Affiliations:** 1 Biomedical Magnetic Resonance Research Group, Louvain Drug Research Institute, Université catholique de Louvain (UCLouvain), Avenue Mounier 73.08, B –, 1200 Brussels, Belgium; 2 Department of Dermatology, Melanoma Clinic, King Albert II Institute, St Luc Hospital, Université catholique de Louvain (UCLouvain), Brussels, Belgium; 3 Nuclear and Electron Spin Technologies (NEST) Platform, Louvain Drug Research Institute, |Université catholique de Louvain (UCLouvain), Brussels, Belgium; 4 Department of Oncology, Melanoma Clinic, King Albert II Institute, St Luc Hospital, Université catholique de Louvain (UCLouvain), Brussels, Belgium

**Keywords:** EPR, ESR, Cancer, Melanoma, *In vivo*, Clinical EPR, Melanin, Mice, Patients, Low frequency

## Abstract

The incidence of melanoma is continuously increasing over time. Melanoma is the most aggressive skin cancer, significantly reducing quality of life and survival rates of patients at advanced stages. Therefore, early diagnosis remains the key to change the prognosis of patients with melanoma. In this context, advanced technologies are under evaluation to increase the accuracy of the diagnostic, to better characterize the lesions and visualize their possible invasiveness in the epidermis. Among the innovative methods, because melanin is paramagnetic, clinical low frequency electron paramagnetic resonance (EPR) that characterizes the melanin content in the lesion has the potential to be an adjunct diagnostic method of melanoma. In this review, we first summarize the challenges faced by dermatologists and oncologists in melanoma diagnostic and management. We also provide a historical perspective on melanin detection with a focus on EPR spectroscopy/imaging of melanomas. We describe key elements that allow EPR to move from *in vitro* studies to *in vivo* and finally to patients for melanoma studies. Finally, we provide a critical view on challenges to meet to make EPR operational in the clinic to characterize pigmented lesions.

## Introduction

Melanoma incidence is continuously increasing over the last decades. While early (thin) melanomas are cured with an excellent survival prognosis by performing a simple surgical excision, advanced (thick) melanomas show limited survival rates due to their high metastatic rate. Even if immunotherapy and targeted therapies have significantly extended survival, the success of these therapies are effective only in a fraction of patients. Therefore, the early diagnosis remains the key to improve the chance to cure melanoma patients. In this context, advanced technologies are under evaluation to increase the accuracy of the diagnostic, to better characterize the lesions and visualize their possible invasiveness in the epidermis. Among the innovative methods, clinical low frequency electron paramagnetic resonance (EPR) that characterizes the melanin content in the lesion has the potential to be an adjunct diagnostic method of melanoma. To understand the potential added value of EPR, this paper briefly summarizes current practices in skin melanoma diagnosis and challenges faced by the dermatologists for patient management before describing the potential impact of EPR as a diagnostic aid to characterize skin melanomas.

## Practice and Challenges in Skin Melanoma Diagnosis

Key elements in the diagnosis of melanoma include the patient’s history, physical examination, and diagnostic aids [1]. Alarming signs well known from the general public are generally referred to the ABCDE patterns, where A stands for asymmetry of the lesion, B for irregular borders, C for a variety of colors, D for diameter usually larger than 6 mm, and E for an evolving lesion over time. The “ugly duckling” is another warning sign of melanoma. This feature is based on the concept that most normal moles (or nevi) would resemble each other, while melanomas stand out like ugly ducklings in comparison. Melanomas may actually present different growth patterns [1]. Superficial spreading melanoma (SSM) generally presents a rather large lesion with asymmetry, irregular borders, and multiple colors. Nodular melanoma (NM) are smooth or ulcerated nodules often shiny black or red. Lentigo maligna (LM) are generally asymmetric, irregularly pigmented, but often difficult to diagnose. They are present in chronically sun-exposed sites in elderly individuals. Acral lentiginous melanoma (ALM) is a rare form that typically appears on the palms and soles, or sometimes on the nail matrix, presenting as irregular longitudinal lines on the nail.

Diagnosing melanoma by sole visual inspection can be challenging [2]. Nowadays, dermoscopy is the most widely non-invasive technique used in clinical practice to assess skin lesions. Dermoscopy uses a handheld device, with or without polarized light, which allows the observation of skin lesion structures down to the middle dermis. A combination of several features may suggest that a lesion could be a melanoma: atypical pigmented network, blue-white veil, atypical vascular pattern, irregular streaks, irregular and asymmetric pigmentation, irregularly distributed dots/globules, regression structures. Dermoscopy shows a sensitivity of 90% and a specificity of 42% for melanoma diagnosis [3]. It should be emphasized that the sensitivity and specificity are strongly operator-dependent, depending mainly on the training received [4]. In addition to dermoscopy, several advanced diagnostic technologies have been proposed such as reflectance confocal microscopy (RCM). RCM is a non-invasive technique for examining the different layers of the skin. The reflection of a near-infrared light from a diode laser that passes between the cellular structures of the skin gives a two-dimensional gray image using a computer software [5]. RCM assesses the cellular details of skin lesions down to the superficial dermis. RCM is very effective for distinguishing benign from malignant melanocytic lesions with a sensitivity of 92% and specificity of 70% when used by trained dermatologists [6]. Optical coherence tomography (OCT) magnifies the surface of a skin lesion to the level of that seen using a microscope using near-infrared light. OCT has also been shown to be useful in tumor margin delineation and is, thus, useful in preoperative treatment planning [7]. However, a systematic review concluded in lack of sufficient data for the use of OCT as a melanoma diagnostic technique [8].

**Fig. 1:**
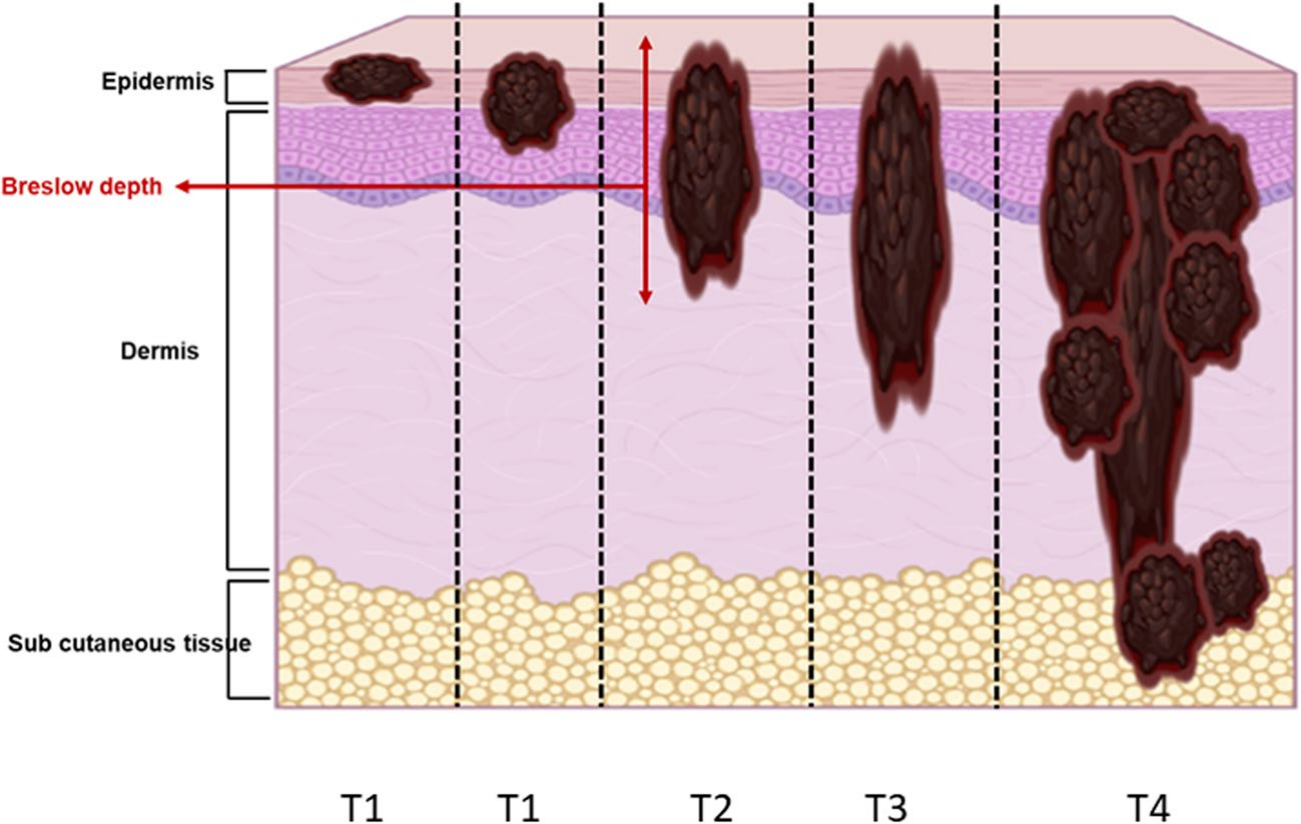
Illustration of different patterns of skin invasion by melanoma cells. Skin melanoma staging is based on the TNM (tumor, node, metastasis) characterization. The tumor category T is determined based on Breslow tumor thickness and the presence or absence of ulceration. The stage of the skin lesion is dependent on the tumor thickness, with T1, T2, T3, and T4 lesions presenting a thickness smaller than 1 mm, between 1 and 2 mm, between 2 and 4 mm, and larger than 4 mm, respectively. Melanomas with a Breslow thickness lower than 0.8 mm have an excellent prognostic. Thicker the melanomas, higher the risk of migration of melanoma cells to lymph nodes (N) and higher the risk of distant metastases (M)

When a lesion is suspected of melanoma, a decision is taken to excise the lesion with minimal margins. The histological analysis of the lesion will confirm the diagnostic benign/malignant. The primary tumor thickness (known as Breslow index, measured from the top of the tumor to the deepest tumor cells, Fig. 1) and presence of ulceration are the two major prognostic factors for survival and skin melanoma staging [9]. When the Breslow depth is smaller than 0.8 mm and the tumor is not ulcerated, a second surgery is performed to enlarge the margins (1 cm) of resection. When the Breslow thickness is greater than 0.8 mm and/or the melanoma is ulcerated, the same second surgery is performed (margins of 1 or 2 cm according to the Breslow thickness) but simultaneous sentinel lymph node biopsy is associated in most cases. Indeed, regional lymph nodes represent the first sites of metastases in patients with melanoma.

While advanced technologies described previously are valuable tools for early melanoma diagnosis, it should be emphasized that none is able to give an accurate *in vivo* estimation of the Breslow thickness. Ultra-high frequency ultrasound (HFUS) has been reported to be able to determine a Breslow thickness comparable to histological infiltration assessment on skin biopsies [7]. However, while HFUS may define the thickness of a lesion, this method has no specificity regarding the nature of the lesion.

## EPR of Melanin: a Historical Perspective

Melanin is a naturally occurring pigment found in animals, plants, and microorganisms. It is responsible for the coloration of skin, hair, eyes, and feathers in animals. In humans, melanin is produced by specialized cells called melanocytes, which are located in the basal layer of epidermis, hair follicles, and eyes [10]. Melanin is a mixture of polymers derived from the oxidation of L-tyrosine by the melanogenic enzyme tyrosinase [11]. The melanogenic process can lead to two major forms: insoluble brown to black pigments named eumelanin and alkali-soluble yellow to reddish-brown pigments termed pheomelanin. Eumelanin is the predominant form of melanin in human skin and hair, and responsible for the dark tone in humans, while pheomelanin is responsible for the red and yellow colors in hair and skin [12]. Interestingly, melanin polymers contain stabilized semi-quinone free radicals in their structure [13, 14].

**Fig. 2:**
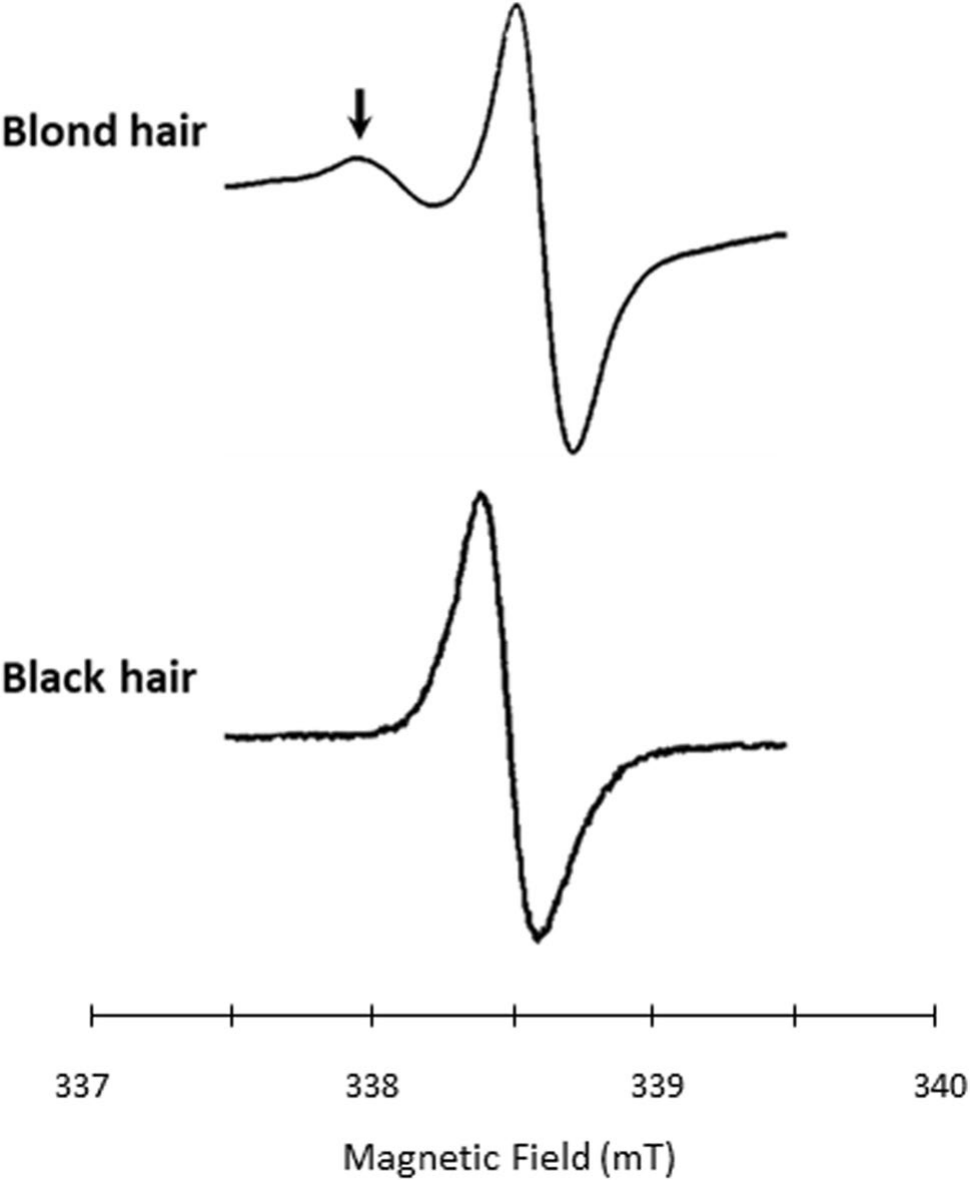
Representative EPR spectra (9 GHz) of natural eu- and pheomelanins detected in human hair samples. Bottom: spectrum of eumelanin signal recorded from black hairs (semiquinone free radicals). Top: spectrum of pheomelanin from blond hairs (note the presence of a shoulder at low field due to the presence of o-semiquinone-imine free radicals)

Electron paramagnetic resonance (EPR) or, equivalently, electron spin resonance (ESR) is a magnetic resonance technology that characterizes paramagnetic species which contain unpaired electrons (free radicals and paramagnetic transition metal ions). It is based on the absorption of the electromagnetic radiation by a sample placed in a magnetic field. The most classical continuous-wave (CW) EPR spectrometers irradiate the sample at a constant frequency of 9 GHz (X-Band) with a magnetic field sweeping around 0.3 Tesla. The first scientific description of an EPR signal linked to the presence of melanin appeared in Nature in 1954, where the EPR signal observed in some biological materials was attributed to free radicals trapped in the melanin pigment [15]. It was then confirmed that melanin from different sources (sepia, human hair) produced remarkably identical EPR signals [16]. The presence of an EPR signal coming from melanoma models was first published by Nebert in 1963 [17] followed by the description of an EPR signal in paraffin-embedded ocular melanomas coming from human biopsies [18]. In this last study, the authors found a positive correlation between the EPR signal intensity and the number of melanin granules in the slides. Later, it was observed that both forms of melanin, eumelanin and pheomelanin, produce two distinct EPR spectra [13] (Fig. 2). While the EPR spectrum of eumelanin presents a single line that is due to the semiquinone radical, semiquinonimine free radicals derived from cysteinyL-dopa in pheomelanin present a distinct feature due to the interaction with a nitrogen atom (Fig. 2) [13]. The single EPR line (with *g* = 2.005) recorded from human melanoma was ascribed to eumelanin [13]. While melanotic melanomas produced pure eumelanin, amelanotic variants are devoid of melanin pigment and melanosomes [19]. Of note, the incidence of amelanotic and hypomelanotic melanomas is very low, with less than 2% of newly diagnosed melanomas each year [20]. Therefore, for the rest of this review paper, we will focus on eumelanin that is the main pigment present in the very large majority of melanomas.

Several factors may contribute to variations in the EPR signal of melanin, as reviewed by Sarna and Plonka in 2005 [11]. It includes the reactivity of melanin towards UV radiation as well as the effect of pH and temperature on the EPR signal of melanin. In particular, it has been shown that the EPR signal intensity from melanin is influenced by the presence of metal ions which can be chelated by melanin. While the melanin EPR signal might be attenuated by paramagnetic ions like copper (II) and iron (III), it could be enhanced by diamagnetic metal ions like zinc (II) or cadmium (II). It is important to note that most studies focused on chelation properties of melanin were performed under conditions that do not mimic physiological conditions such as cryogenic temperature [21]. The physiological variation in the concentration of metal ions present in melanocytic and melanoma cells and its impact on the EPR signal of melanin in those cells remain largely unexplored so far.

## EPR Imaging of Skin Melanoma Biopsies

Using magnetic field gradients, EPR imaging provides two- or three-dimensional images of the distribution of paramagnetic species in a sample. The first study that described an attempt to map melanin in tumors using EPR imaging was published in 1990 by Katsuda et al. [22]. Using a pin-hole cavity resonator in a X-Band (9 GHz) system, these authors measured the EPR signal in samples of melanin. They provided a figure with a rough distribution of the melanin present in a frozen solid slide of a melanoma (kept at −39 °C) that was removed from the back of the mice implanted with B16 melanoma cells.

**Fig. 3:**
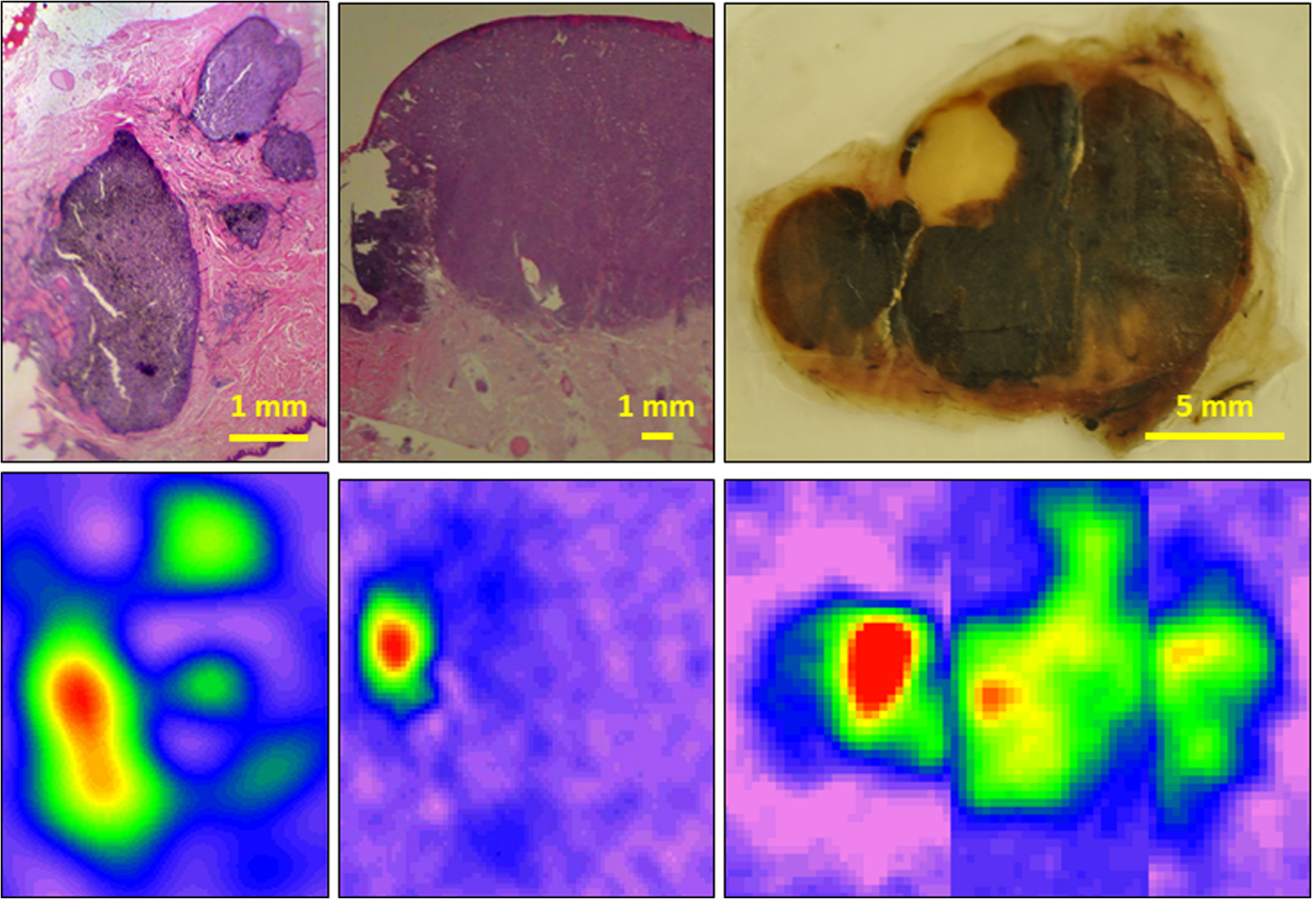
Comparison between anatomopathological samples (top row) and their corresponding EPR images obtained on human melanoma samples with a 9 GHz spectrometer. Adapted from results described in studies [23, 26]

Because the thickness of the skin melanoma (Breslow depth) is the most crucial prognostic factor in patient management, there is a major interest for a method able to provide a map of the distribution of melanoma cells in tissues. A crucial step in this research was achieved with the publication of Vanea et al. [23]. Using a commercial 9 GHz EPR imaging system, 2D and 3D images were obtained in freeze-dried samples coming from B16 melanomas grown in mice. 3D images were also obtained from melanoma metastases that colonized the lungs. High resolution images were obtained from a paraffin-embedded melanoma metastasis with irregular clusters of melanocytes present in the derma of a patient (Fig. 3). 2D images were also provided from fresh wet melanoma tissues. Finally, as a proof-of-principle, the first EPR image of melanin was recorded *in vivo* at a microwave frequency of 1 GHz using a mouse head coil. This was achieved on a very large tumor implanted on the head of a mouse. This localization was selected to minimize any motion artifact. Overall, this study provided the first EPR detection of an endogenous radical *in vivo* [23].

As part of the development of EPR imaging to characterize melanomas, important incremental studies were carried out during the PhD thesis of Quentin Godechal. In a first study [24], he compared the EPR signal with the extension of primary tumors and lung metastases of a pigmented melanoma model expressing luciferase. In practice, he measured light emission by bioluminescence *in vivo* and quantified the EPR signal on freeze-dried samples after excision. He observed a direct linear correlation between the EPR intensity and the bioluminescence intensity, suggesting that EPR can be a marker of stage or invasiveness [24]. Next, he analyzed the capabilities of EPR imaging to provide a real distribution of melanin pigment in different samples [25]. He demonstrated that EPR imaging (using a commercial 9 GHz EPR system) provided accurate images of synthetic samples (phantoms of sucrose loaded with melanin), both in terms of shape and size, with errors always lower than 10% compared to the real area [25]. The ability of EPR imaging to accurately map melanomas was dependent on the concentration of melanin in the sample, which was proportional to the growth stage of the tumor and the consequent signal-to-noise ratio (SNR) of the EPR signal. The tumor stage is dependent on the tumor thickness, with T1, T2, T3, and T4 lesions presenting a thickness smaller than 1 mm, between 1 and 2 mm, between 2 and 4 mm, and larger than 4 mm, respectively. The EPR signal was more intense in higher-grade (T4 and T3) than in lower-grade (T2 and T1) histological samples coming from human tumors. Accurate images of melanin distribution were obtained for higher stage melanoma samples [25]. In a third study [26], he used EPR imaging as a tool to map the concentration of melanin inside *ex vivo* human pigmented (*n*=3) and nonpigmented melanomas (*n*=3) and correlated the EPR cartography with histology. He obtained accurate mappings of the melanin inside pigmented human melanoma samples. The signal intensity observed on the EPR images correlated with the concentration of melanin within the tumors, visible on the histologic sections. The EPR images remarkably overlaid over the histological sections (Fig. 3). In contrast, no EPR signal coming from melanin was observed from non-pigmented melanomas confirming that the application of EPR to characterize melanomas should be restricted to pigmented melanomas, the most prevalent form occurring in patients [26].

### *In Vivo* EPR of Melanin in Melanomas: from Mice to Patients

To move from *in vitro* to *in vivo* studies, there was a need to decrease the operating frequency. The classical EPR spectrometers operate at 9 GHz. At this frequency, the analysis on biological samples requires operating on dehydrated materials (for example, freeze-dried samples) or very thin material (suspension of cells in capillaries or thin slices (less than 1 mm thickness) of tissues). To apply EPR *in vivo*, it is possible to use 1 GHz EPR spectrometers/imagers offering the possibility to explore up to 1 cm depth (which is optimal for exploring the skin) or systems operating at lower frequency. However, we should keep in mind that the decrease in frequency is at the cost of a dramatic decrease in sensitivity of detection, a crucial issue when trying to detect the very weak signal coming from an endogenous free radical.

**Fig. 4:**
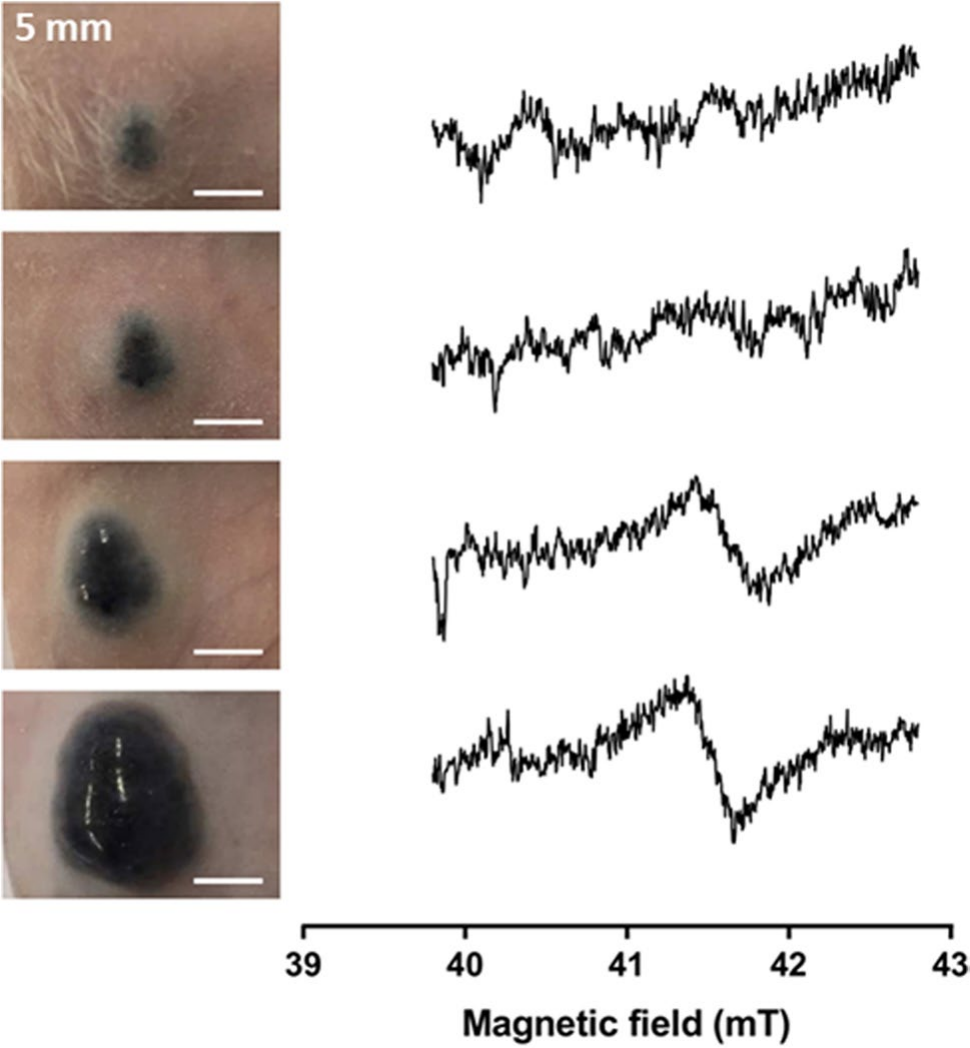
Influence of tumor growth (B16 melanoma model) on the EPR signal recorded *in vivo* in mice using a clinical EPR spectrometer operating at 1 GHz. Adapted from results described in studies [32]

As mentioned earlier, the first direct, non-invasive experimental *in vivo* detection of melanin was described by Vanea et al. [23]. Since that time, the construction of whole-body clinical EPR systems and adapted resonators [27, 28] has opened the possibility of applying EPR to human subjects [29–31]. Using a 1 GHz clinical EPR spectrometer (ClinEPR) installed in Brussels, Desmet et al. checked the feasibility to detect melanin *in vivo* in melanoma tumor models implanted in mice [32]. She found a significant correlation between the tumor size of pigmented B16 melanoma (Fig. 4) while no EPR signal was recorded from achromic non-pigmented WM2664 melanomas. These developments paved the way for initiating clinical trials in Brussels for melanoma characterization by EPR spectrometry.

The initiation of the first clinical trial in Brussels to detect melanin in humans was actually strongly influenced by a cornerstone study carried out by Cesareo et al. on human biopsies [33]. These authors performed an EPR analysis *in vitro* (9 GHz) on paraffin-embedded human melanoma (*n*=52) and nevi (*n*=60). They observed that the EPR signal in nevi was significantly lower than that in melanomas and found that the EPR signal was significantly different between low Breslow’s and high Breslow’s depth melanomas. Finally, an ROC (receiver operating characteristic) analysis using ESR data discriminated melanomas sections from nevi sections with up to 90% accuracy [33].

**Fig. 5:**
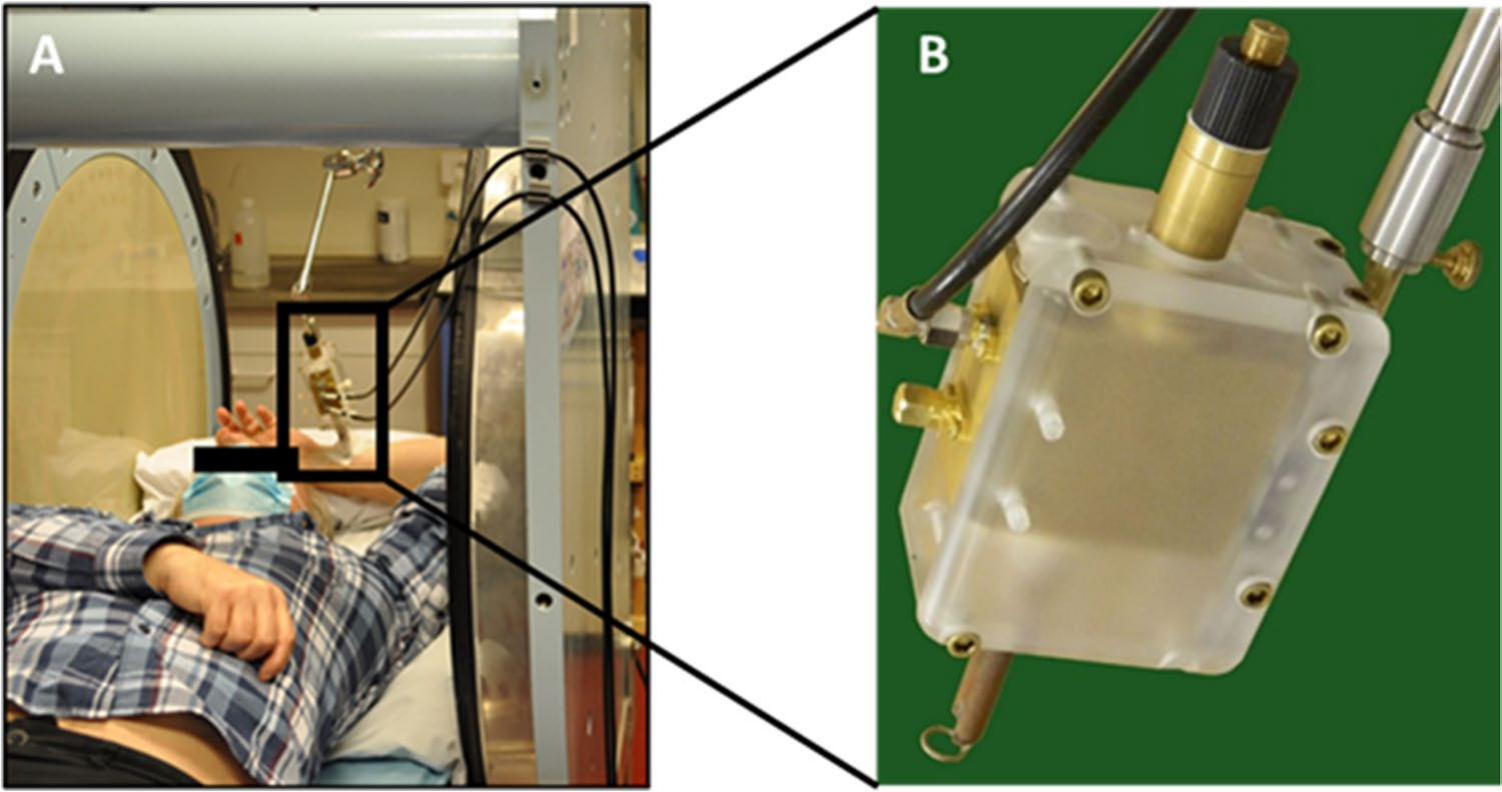
Experimental set-up used in the clinical studies in Brussels on lesions suspect of melanoma (from studies described in [34]. **A** Person installed in low field magnet with a surface coil put at the surface of the analyzed lesion on the arm. **B** Focus on the surface coil resonator

**Fig. 6:**
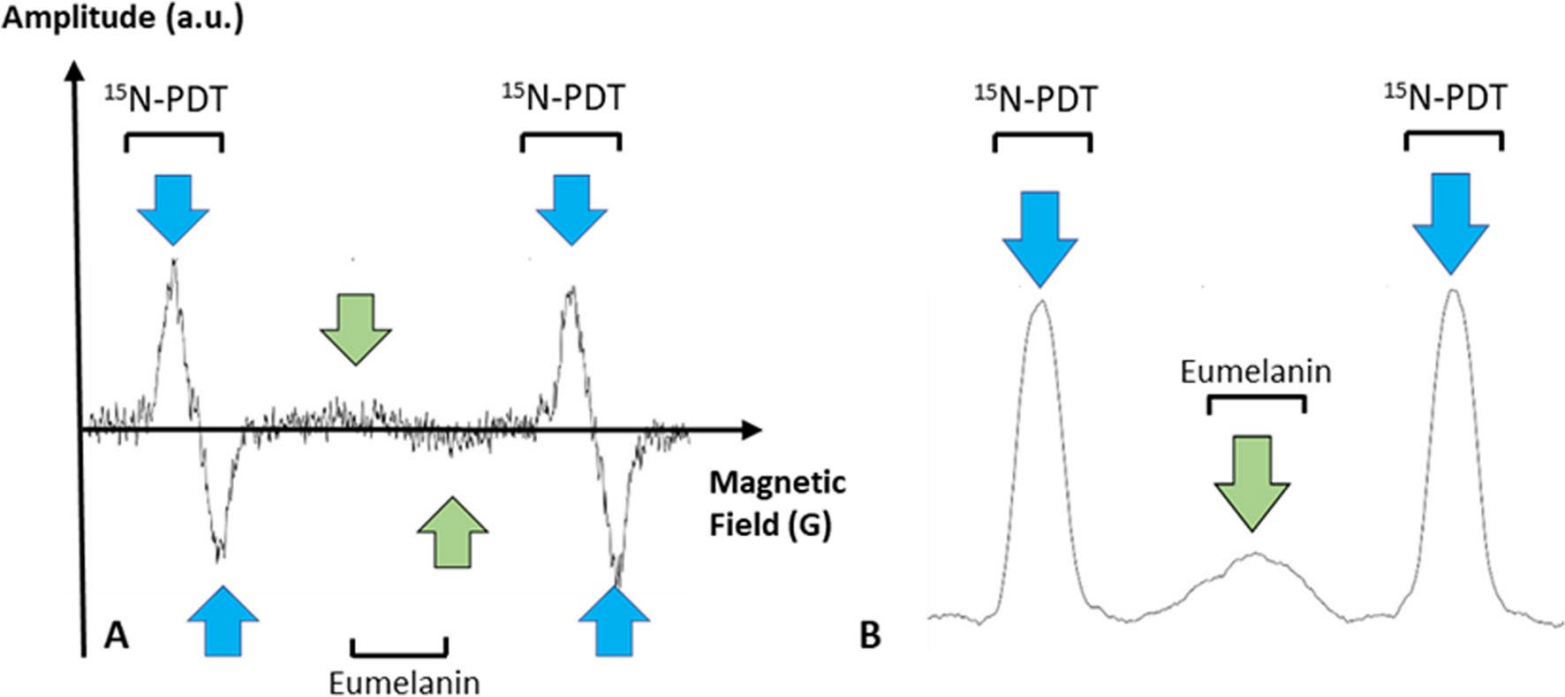
Data recorded noninvasively from a melanoma lesion in a patient enrolled in the clinical EPR study described in [34]. **A** Average of first derivative EPR spectra recorded from the skin. The melanin signal is in the center (highlighted by green arrows). For field positioning and quantification, a reference spectrum is taken using a sample of ^15^N-perdeuterated nitroxide (^15^N-PDT) fixed along the axis of the resonator. The signal of the reference appears as a doublet (highlighted by the blue arrows). **B** First integration of this signal

**Fig. 7:**
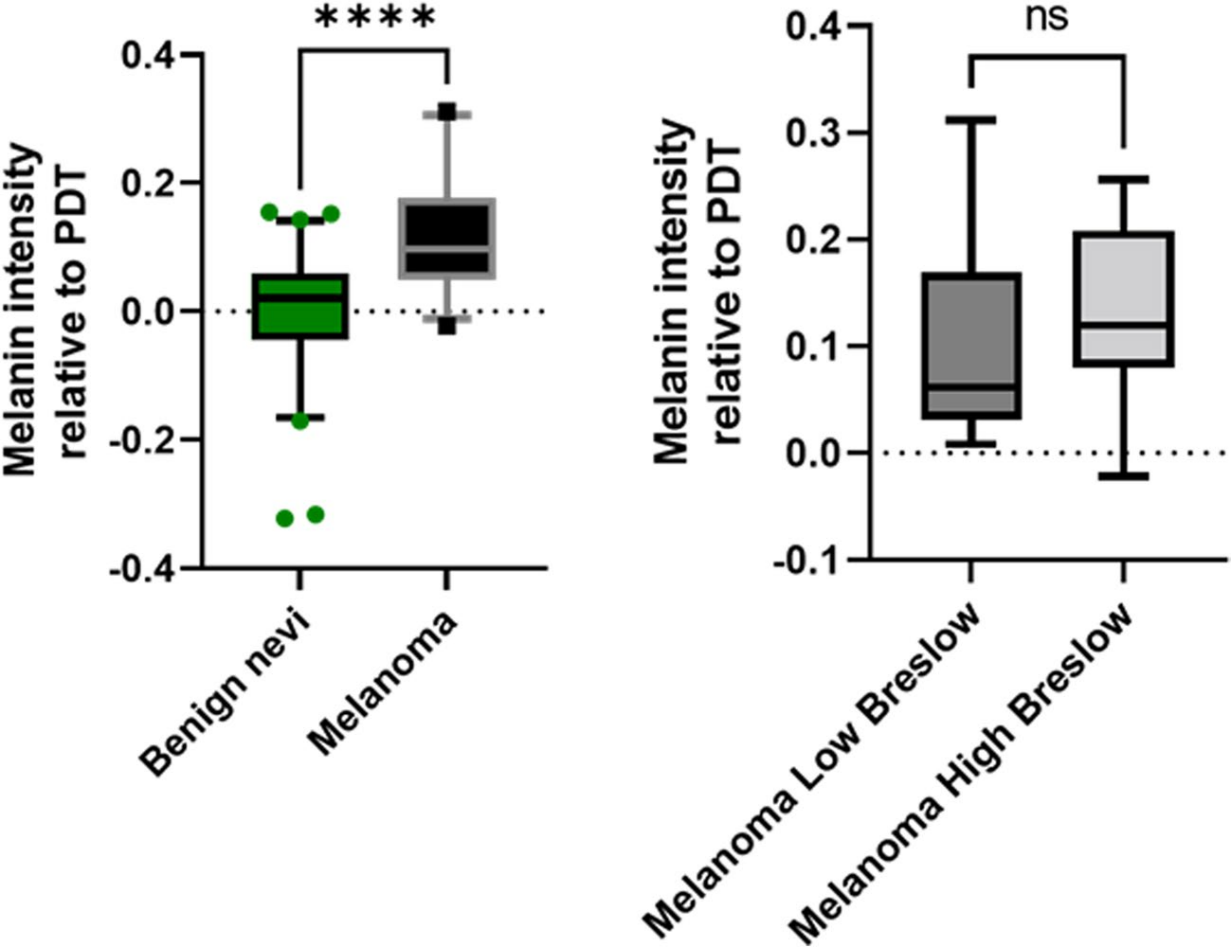
Main results from the clinical EPR study described in [34]. Left: the EPR signal of melanin was significantly higher in melanoma lesions (*n*=26) compared to the signal recorded in benign atypical nevi (*n*=62) (****, *p* < 0.0001). Right: a trend toward a higher signal intensity (though not significant) was observed in high Breslow depth melanomas than in low Breslow lesions

It was an open question whether this observation done *in vitro* by Cesareo was translatable noninvasively in patients using clinical EPR. Therefore, the ambition of the first clinical trial in this field done by Mignion et al. [34] was to explore the feasibility of noninvasive detection of melanin in suspect pigmented skin lesions (Fig. 5), to evaluate the value of the technology to discriminate between benign and malignant lesions, and to compare the EPR signal recorded with the Breslow depth measured by histopathology. The transition from animal studies to application on patients imposed to deal with new challenges ahead. While animals are anesthetized and can be kept for a long time in the EPR magnet to accumulate EPR spectra, clinical measurements should be performed rapidly and efficiently in humans. Moreover, as lesions could be located in body areas prone to motion, it was necessary to use rapid acquisitions compatible with requests for immobility and, sometimes, breath holding. In practice, the authors acquired multiple series of two scans of 5 s with relaxing periods between the EPR acquisitions. The resulting EPR spectra presented an extremely low signal-to-noise ratio. This prompted the researchers to use a ^15^N-nitroxide (^15^N-PDT) in the coil as a reference signal to assign magnetic field positioning and quantitative analysis, the peak of melanin appearing between the two external lines of PDT (Fig. 6). In their recent publication, the authors actually reported the results of two clinical studies [34]. The first one performed on 45 healthy volunteers presented different skin phototypes (classification of the skin by its pigmentation and reaction to sunlight exposure). The second one on patients with skin lesions suspect of melanoma (*n* = 100) requiring surgical resection. The patients enrolled in this study came for the EPR analysis just before the surgical excision, and EPR results were compared with histopathology results. The main result of the study on healthy volunteers revealed that the method was not sensitive enough to measure differences in melanin content due to changes in skin pigmentation. This indicated also that pigmentation could not play a role in the evaluation of melanin content in skin lesions. In patients, 92% of the spectra were analyzable. The EPR signal of melanin was significantly higher (*p* < 0.0001) in melanoma lesions than that in benign atypical nevi (Fig. 7) [34], confirming in a noninvasive way the results obtained by Cesareo who used high frequency EPR on biopsies. A trend toward a higher signal intensity (though not significant) was observed in high Breslow depth melanomas (a marker of skin invasion) than in low Breslow lesions (Fig. 7).

### Filling the Present Research Gaps for a Successful Clinical Implementation

The main achievement of the clinical study on suspect melanoma lesions [34] was the noninvasive detection of an endogenous free radical in human beings by EPR. So far, other clinical studies with low-frequency EPR required administration of an exogenous paramagnetic sensor, for example to measure tissue oxygenation [29–31]. Other studies recorded radio-induced EPR signal in teeth after an external high irradiation dose [35, 36]. The study of Mignion et al. suggests that clinical EPR could be used as an adjunct aid to the diagnosis of suspect pigmented lesions [34]. However, as already emphasized, the SNR of the EPR signal recorded in skin lesions was extremely low. For future and successful clinical implementation, there is unquestionably a room to increase the sensitivity of detection. A possible option would be to use new acquisition modes, for example multi-harmonics EPR [37] or rapid-scan EPR [38, 39] that enable the use of higher modulation amplitude and power. The clinical L-Band EPR spectrometer in Brussels used in this first clinical trial on melanoma patients has just been upgraded with capability to use higher power, higher modulation amplitude and capability of multi-harmonic analysis. In a work in progress using multi-harmonic detection, preliminary results obtained on melanoma implanted in mice indicate a large EPR signal amplification (publication in preparation) opening the way to start a new large clinical trial in patients presenting suspect pigmented lesions. Of note, other improvements in instrumentation, such as the development of S-Band (2–3 GHz) bridges compatible with clinical EPR spectrometers, could be another option to boost the sensitivity. This higher frequency would increase the SNR while allowing detection of melanin up to a 4 mm skin penetration depth [40]. Minimizing motion artifacts during EPR registration could also facilitate the registration of EPR spectra. In this regard, optimizing flexible resonators designed for skin measurements would be very useful [41]. In case of increase in sensitivity by an order of magnitude to detect melanin in skin, it would be reasonable to think about using 1D or 2D gradients to record the distribution of melanin in the superficial layer of skin (a few millimeters), even with a low spatial resolution. As the management of patients is based on the estimate of the Breslow depth, the availability of a noninvasive technology able to discriminate melanocytic skin lesions with 0.5, 1, 2, 3, or 4 mm thickness would constitute an important breakthrough for dermatologists. We may even dream of miniaturized EPR imaging systems that could be used at the surface of the skin to render the method more operational in multiple centers that are not experts in EPR.

In addition to instrumental developments, the recent progress obtained in the first clinical trial on melanoma patients could stimulate more fundamental research. While both studies of Cesareo (*in vitro*, 122 lesions) and Mignion (*in vivo*, 100 lesions) provided consistent results, the reason why the EPR signal intensity is higher in melanoma compared to nevi remains speculative. This could stimulate a clinical trial on human biopsies to study melanin polymer organization, melanogenesis, or melanin degradation in nevi and in skin melanomas [11, 12, 42, 43]. Knowing also that the EPR signal intensity could be affected by the presence of ions such as iron (III), copper (II), and zinc (II) [21], it would make sense to quantify these metals in a large set of nevi and skin melanomas. This would fine-tune and validate the interpretation of EPR data obtained in patients.
